# Effects of Intracanal Irrigant MTAD Combined with Nisin at Sub-Minimum Inhibitory Concentration Levels on *Enterococcus faecalis* Growth and the Expression of Pathogenic Genes

**DOI:** 10.1371/journal.pone.0090235

**Published:** 2014-03-06

**Authors:** Zhongchun Tong, Lijia Huang, Junqi Ling, Xueli Mao, Yang Ning, Dongmei Deng

**Affiliations:** 1 Department of Operative Dentistry and Endodontics, Guanghua School of Stomatology, Sun Yat-sen University, Guangzhou, Guangdong, China; 2 Guangdong Provincial Key Laboratory of Stomatology, Sun Yat-sen University, Guangzhou, Guangdong, China; 3 Department of Periodontology, Guanghua School of Stomatology, Sun Yat-sen University, Guangzhou, Guangdong, China; 4 Department of Cariology Endodontology Pedodontology, Academic Centre for Dentistry Amsterdam (ACTA), Universiteit van Amsterdam and Vrije Universiteit, Amsterdam, The Netherlands; University of Padova, Medical School, Italy

## Abstract

Exposure to antibiotics is considered to be the major driver in the selection of antibiotic-resistant bacteria and may induce diverse biological responses in bacteria. MTAD is a common intracanal irrigant, but its bactericidal activity remains to be improved. Previous studies have indicated that the antimicrobial peptide nisin can significantly improve the bactericidal activity of MTAD against *Enterococcus faecalis*. However, the effects of MTAD and its modification at sub-minimum inhibitory concentration (sub-MIC) levels on *Enterococcus faecalis* growth and the expression of pathogenic genes still need to be explored. In this study, the results of post-antibiotic effects (PAE) and post-antibiotic sub-MIC effects (PASME) showed that MTADN (nisin in combination with MTAD) had the best post-antibiotic effect. *E. faecalis* after challenge with MTAD was less sensitive to alkaline solutions compared with MTAN (nisin in place of doxycycline in MTAD) and MTADN. *E. faecalis* induced with sub-MIC of MTAD generated resistance to the higher concentration, but induction of *E. faecalis* with MTAN did not cause resistance to higher concentrations. Furthermore, real-time polymerase chain reaction (RT-PCR) showed that the stress caused by sub-MIC exposure to MTAD, MTAN, or MTADN resulted in up- or down-regulation of nine stress genes and four virulence-associated genes in *E. faecalis* and resulted in different stress states. These findings suggested that nisin improved the post-antibacterial effect of MTAD at sub-MIC levels and has considerable potential for use as a modification of MTAD.

## Introduction

Enterococci are now among the leading causative agents of nosocomial infections due to their high levels of resistance to many antibiotics [1]. *Enterococcus faecalis*, a common enterococcal species, is a natural commensal of the gastrointestinal tract of humans and animals. However, it can also become an opportunistic pathogen involved in urinary tract infections, surgical wound infections, bacteremia, bacterial endocarditis, and chronic periapical periodontitis [2], [3]. *E. faecalis* is a robust microorganism that can withstand various harsh conditions, such as sodium dodecyl sulfate, bile salts, hyperosmolarity, heat, ethanol, hydrogen peroxide, acidity, and alkalinity [4]. *E. faecalis* is typically recovered from the root canals of teeth with post-treatment disease, in which the occurrence of *E. faecalis* ranges from 24% to 77% [5]. Thus, it is important to consider the effects of intracanal irrigants on *E. faecalis* during clinical application in therapies of chronic periapical periodontitis.

MTAD, a common intracanal irrigant, consists of 3% doxycycline, 4.5% citric acid, and 0.5% polysorbate 80 detergent and is used to remove pathogenic bacteria and smear layers during root canal procedures [6]. MTAD has many advantages in root canal irrigation, but its bactericidal activity remains to be improved upon as its antibacterial effect has been largely attributed to doxycycline, a tetracycline that is bacteriostatic rather than bactericidal [7]. In a previous study, nisin, an antibacterial peptide, was used as a substitute for or in combination with doxycycline in MTAD to improve its bactericidal activity. We found that MTADN (nisin combined with MTAD) had the best antibacterial activity in evaluating the effects of MTAD, MTAN (nisin in place of doxycycline in MTAD), and MTADN on *E. faecalis* during exponential growth phase and stress states [8], [9]. However, concentrations of the drugs gradually decreased and reached sub-minimum inhibitory concentrations (sub-MIC) due to rinsing and dilution during the root canal procedure, and the potential effects of sub-MIC levels of the drugs on *E. faecalis* should be further studied. Furthermore, in the treatment of periapical periodontitis, alkaline calcium hydroxide is generally used as an intracanal dressing to inhibit pathogens in the root canal after the root canal is washed with irrigants. During this time, the pathogens will be challenged in an alkaline environment. Thus, *E. faecalis* exposure to alkaline conditions after irrigant treatment should be further evaluated.

Antibiotics at lethal concentrations seldom occur outside therapeutic applications; however, bacteria frequently confront sub-MIC levels of antibiotics in the environment and the host following therapy. The pharmacodynamic activities of antimicrobial agents can significantly differ in variables in addition to their minimum inhibitory concentrations (MIC) and minimum bactericidal concentrations (MBC) for pathogens. Post-antibiotic effects (PAE) and post-antibiotic sub-MIC effects (PASME) are two such examples of variables that possess differences and similarities, which may have an important effect on the emergence of microorganisms that are resistant to conventionally used agents [10]. Among the pharmacodynamic variables, PAE and PASME represent the persistence and after-effects of antimicrobial agents on pathogens, respectively [11]. Furthermore, antibacterial agents at sub-MIC levels may induce greater bacterial resistance and stress responses and result in the enhancement of bacterial pathogenicity [12]. Thus, in the present study, the effects of sub-MIC levels of MTAD and their modifications on *E. faecalis* were evaluated by determining the PAE, PASME, drug resistance, and alkaline challenge. Real-time polymerase chain reaction (RT-PCR) was used to evaluate the expression of stress genes, *dnaK*, *groEL*, *ace*, *ctsR*, *clpC*, *clpE*, *clpP*, *clpX*, and *gls24*, and of virulence-associated genes, *cylB*, *efaA*, *gelE*, and the *sprE* gene in *E. faecalis.*

## Materials and Methods

### Bacterial culture and preparation of antibiotics

*Enterococcus faecalis* ATCC 29212 was used in this study. *E. faecalis* was routinely streaked on brain heart infusion (BHI) agar (1.5% w/v, Difco, USA) from frozen stocks and cultured aerobically at 37°C for 24 h. A single bacterial colony was inoculated into 5 ml of BHI broth and grown overnight. Next, aliquots (500 µl) of overnight cells were added into 50 ml of BHI broth and cultured for 12 h. The number of viable cells was quantified by plate count each hour and was used to plot *E. faecalis* growth curves. MTAD, MTAN, and MTADN were prepared according to a previous procedure [8], [9]. MTAD consisted of 3% doxycycline, 4.25% citric acid, and 0.5% polysorbate 80 detergent (Sigma-Aldrich Co, St Louis, MO), and MTAN contained 3% nisin (a commercial preparation, 1000 IU/mg; Sigma-Aldrich), 4.25% citric acid, and 0.5% polysorbate 80 detergent. MTAD in combination with 3% nisin was referred to as MTADN.

### PAE and PASME assay

*E. faecalis* was grown to a concentration of approximately 10^9^ colony-forming units (CFU/ml) according to growth curves and was prepared for use in PAE and PASME assays. Aliquots (1 ml) of *E. faecalis* culture were transferred into 1.5-ml eppendorf tubes and centrifuged at 6,000 rpm at 4°C for 5 min. The supernatant was discarded, and the bacterial pellet was resuspended and challenged with MTAD for 1 min. Subsequently, the eppendorf tube was immediately centrifuged, and the supernatant was removed. After washing twice with sterile PBS, the bacterial pellet was resuspended in BHI broth. Bacteria that had not been challenged with drugs were used as a positive control. Bacterial survival was evaluated by a viable plate count at 2-hour intervals, and a time vs. bacterial survival curve was plotted. The same assays were performed with MTAN or MTADN. All procedures were independently performed three times on different days, and the means were reported. PAE was defined according to the following formula: PAE = T - C, where T is the time required for viability counts of an antibiotic-exposed culture to increase by 1 log unit above the counts taken immediately after washing, and C is the corresponding time for growth control [13]. For PASME, the bacteria were challenged with MTAD, MTADN, or MTAN for 1 min and were then exposed to 0.1, 0.2, and 0.3 times the MIC of MTAD, MTADN, or MTAN. The MICs of MTAD, MTAN and MTADN against *E. faecalis* ATCC 29212 were obtained from a previous study [8]. Bacterial survival was evaluated by a viable plate count at 2-hour intervals, and time vs. bacterial survival curves were plotted. PASME was obtained using the formula: PASME = T_PA_ - C, where T_PA_ is the time for the previous antibiotic exposed cultures, which had been exposed to different sub-MICs, to increase by 1 log above the counts observed immediately after washing, and C is the corresponding time for the unexposed control [14]. In addition, no bacteria added were referred as the negative controls to monitor absence of contamination.

### Alkaline challenge of *E. faecalis* after antimicrobial treatment

Prior to being challenged with an alkaline solution, *E. faecalis* were initially treated with MTAD, MTAN, or MTADN for 1 min as described in the PAE and PASME assays. Subsequently, *E. faecalis* were immediately exposed to BHI broth at pH 8, pH 9, pH 10, or pH 11. As a positive control, *E. faecalis* without antimicrobial treatment was exposed to BHI broth at the same pH. Bacterial survival was determined by a plate count at 0, 2, 4, 6, 8, 10, 12, 24, 48, 96, 168, and 336 h. To maintain the invariability of the alkaline pH of the broth, different pH and different time points bacterial challenges were performed in 2-ml volumes of freezing tubes (Corning Costar,. Cambridge, MA, USA), which has a tight closure to abstain from the effect of long time exposure to CO_2_ in air on the alkaline pH of the broth. The pH of the broth was assayed to ensure the invariable of pH while bacterial survival was quantified by a plate count in each time point. No bacteria added were referred as the negative controls to monitor the absence of contamination. All of the determinations were repeated three times on different days.

### Assay of *E. faecalis* resistance to antimicrobials

*E. faecalis* has intrinsic resistance to many commonly used antimicrobial agents. *E. faecalis* resistance to MTAD and its modifications was evaluated in this study. Briefly, aliquots (10 µl) of *E. faecalis* in the exponential phase were exposed to 1 ml of 0.5 MIC of MTAD, MTAN, or MTADN at 37°C for 24 h and then streaked onto BHI agar. A single colony was inoculated into 5 ml of BHI broth and grown to the exponential phase. At this phase, the concentrations of *E. faecalis* treated by the three drugs were relatively identical, as assessed by an evaluation of plate count, and were normalized. Subsequently, aliquots (10 µl) of the three bacteria were added into the MTAD, MTAN, or MTADN at the MICs, and the challenges were maintained at 37°C for 24 h. Next, the challenge was continuously performed with 1.5, 2.0, 2.5, and 3.0 times the MIC of MTAD, MTAN, or MTADN. *E. faecalis* survival rates were determined using plate count, and images of the pellet in the eppendorf tube were obtained with a digital camera after treatment with each concentration of antimicrobials.

### Effect of sub-MIC of antimicrobials on the pathogenic genes of *E. faecalis*

A logarithmically growing *E. faecalis* culture was centrifuged and exposed to 0.25 MIC of MTAD, MTAN, and MTADN at 37°C for 1 h. A previous study indicated that 0.5 MIC of nisin alone or in combination with 0.5 MIC of doxycycline could significantly reduce the viable count in an hour; thus, 0.25 MIC was used in this study [8]. An *E. faecalis* culture without challenge with antimicrobials was used as a control. Total RNA was extracted from samples of the three experimental groups and the control group using the TRIzol Max Bacterial RNA Isolation Kit (Invitrogen Corp, Shanghai, China). RNA purity and degradation were confirmed by an evaluation of the A_260_/A_280_ ratio and gel electrophoresis. For cDNA synthesis, 2 µg of RNA was used as a template and was reverse-transcribed using the PrimeScript First Strand cDNA Synthesis Kit (Takara Biotechnology Co., Ltd., Dalian, China). Primers of the pathogenic genes in *E. faecalis* were selected as in Lenz *et al*. (Table 1), and 16S rRNA was used as an internal control [15]. Real-time quantitative PCR (qPCR) assays were performed using SYBR Premix Ex Taq™ (Takara Biotechnology Co., Ltd., Dalian, China) with a Stratagene Mx3000P Real-Time PCR machine (Agilent Technologies, Inc., Santa Clara, United States). The reactions were repeated three times for each gene and consisted of the following components: 10 µl of SYBR Premix Ex Taq TM (2×); 0.4 µl of each primer (10 µM); 2 µl of cDNA; 7.2 µl of deionized water. Next, 2 µl of extracted total RNA in place of cDNA was regarded as the negative control to rule out DNA contamination. The reaction procedure was divided into two stages; the first stage was 95°C for 20 s, and the second stage was 95°C for 5 s, 60°C for 30 s; 40 cycles.

**Table 1:** Primers used to evaluate the expression of genes associated with stress and virulence in *E. faecalis*.

Gene	Primer sequence (5′→3′)	Amplicon length (bp)
*dnaK*	F: CAGTTAACCCTGACGAAG; R: TGACAACGGTGTTACGTC	104 bp
*groEL*	F: CTGTTTCAGTTGCAGCAC; R: CAAATCGGCGAAACAACG	109 bp
*ctsR*	F: CGTTGGATGGTAAAACGTG; R: CGTCAGATTTAATCGAGGC	145 bp
*clpP*	F: GCAATCACTGAGTTTGCC; R: AATCATCTCGCGGTGAAC	109 bp
*clpC*	F: AATACGGGCAAAACGACG; R: AACCTGCTTTAGCACGTG	110 bp
*clpE*	F: CCTAGTAAACCTTGACCAG; R: CTTTCTCGCCAAATGCAAC	125 bp
*clpX*	F: TTCTCGGACTGCTTCATC; R: TAATAACGGGACCGTTCG	154 bp
*gls24*	F: TAACAGTCGATGGCGGCTTT; R: CAGCGACTTGTTTTTTACCAACTTC	105 bp
*efaA*	F: TGGGACAGACCCTCACGAATA; R: CGCCTGTTTCTAAGTTCAAGCC	101 bp
*ace*	F: GGCGACTCAACGTTTGAC; R: TCCAGCCAAATCGCCTAC	100 bp
*gelE*	F: GGAACAGACTGCCGGTTTAG; R: TTCTGGATTAGATGCACCCG	103 bp
*sprE*	F: CCTGTCTGCAAATGCAGAAG; R: CTGCCACTTCTTGTCTTCTG	101 bp
*cylB*	F: ATTGAAGTACGTTGCGCAAG; R: CCCTTAGTTCTACTAGTGTAC	104 bp
23S	F: CCTATCGGCCTCGGCTTAG; R: AGCGAAAGACAGGTGAGAATCC	101 bp

### Statistical analyses

SPSS 18.0 (SPSS Inc., Chicago, IL) was employed for statistical analysis. In the assay of *E. faecalis* resistance to antimicrobials, one-way analysis of variance (ANOVA) and Tukey's honestly significant difference test were used to compare the *E. faecalis* survival rates under the treatment of (1) 0.5 MIC of MTAN, MTAD and MTADN; (2) MIC of MTAN, MTAD and MTADN; (3) 1.5 MIC of MTAN, MTAD and MTADN; (4) 2 MIC of MTAN, MTAD and MTADN; (5) 2.5 MIC of MTAN, MTAD and MTADN; (6) 3 MIC of MTAN, MTAD and MTADN using log10-transformed CFUs counted per milliliter. Furthermore, ANOVA and Tukey's honestly test were used to assess the difference between the relative quantities (RQ) of the gene expressions in *E. faecalis* under different sub-MIC of antimicrobials. A p-value of <0.05 was considered statistically significant.

## Results

### PAE and PASME

In the determination of PAE and PASME, the results were based on the average time obtained from three independent experiments (Table 2). The average PAE of MTAN for *E. faecalis* was significantly shorter compared to the MTAD or MTADN, whereas the average PASME of MTAN at 0.2 times the MIC and 0.3 times the MIC were not determined in a given time and were significantly longer than that of MTAD or MTADN. The average PASME of MTAD, MTADN, and MTAN at 0.1 times the MIC were 7.5, 7, and 6.7 h, respectively, and were not significantly different.

**Table 2:** Post-antibiotic effects and post-antibiotic sub-MIC effects of MTAD, MTAND, and MTAN in *Enterococcus faecalis* ATCC 29212.

Drugs	MIC^a^(dilution fold)	PAE (hours)	PASME (hours)		
			0.1×MIC	0.2×MIC	0.3×MIC
MTAD	1∶8192	3.8±0.6	7.5±1.21	9.05±0.67	10.6±1.8
MTADN	1∶8192	3.6±0.71	7±1.5	8.7±0.94	10.9±2.1
MTAN	1∶32	2.15±0.52	6.7±0.6	∞	∞

PAE and PASME were determined in three independent experiments, and the mean and standard deviations are provided. “∞” indicated that *E. faecalis* was not grown after exposure to 0.2 MIC or 0.3 MIC of MTAN, and 1 log increase did not occur, and thus, PASME was theoretically infinite.

a MIC from Tong et al. [8].

### Alkaline resistance of *E. faecalis*

**Figure 1:**
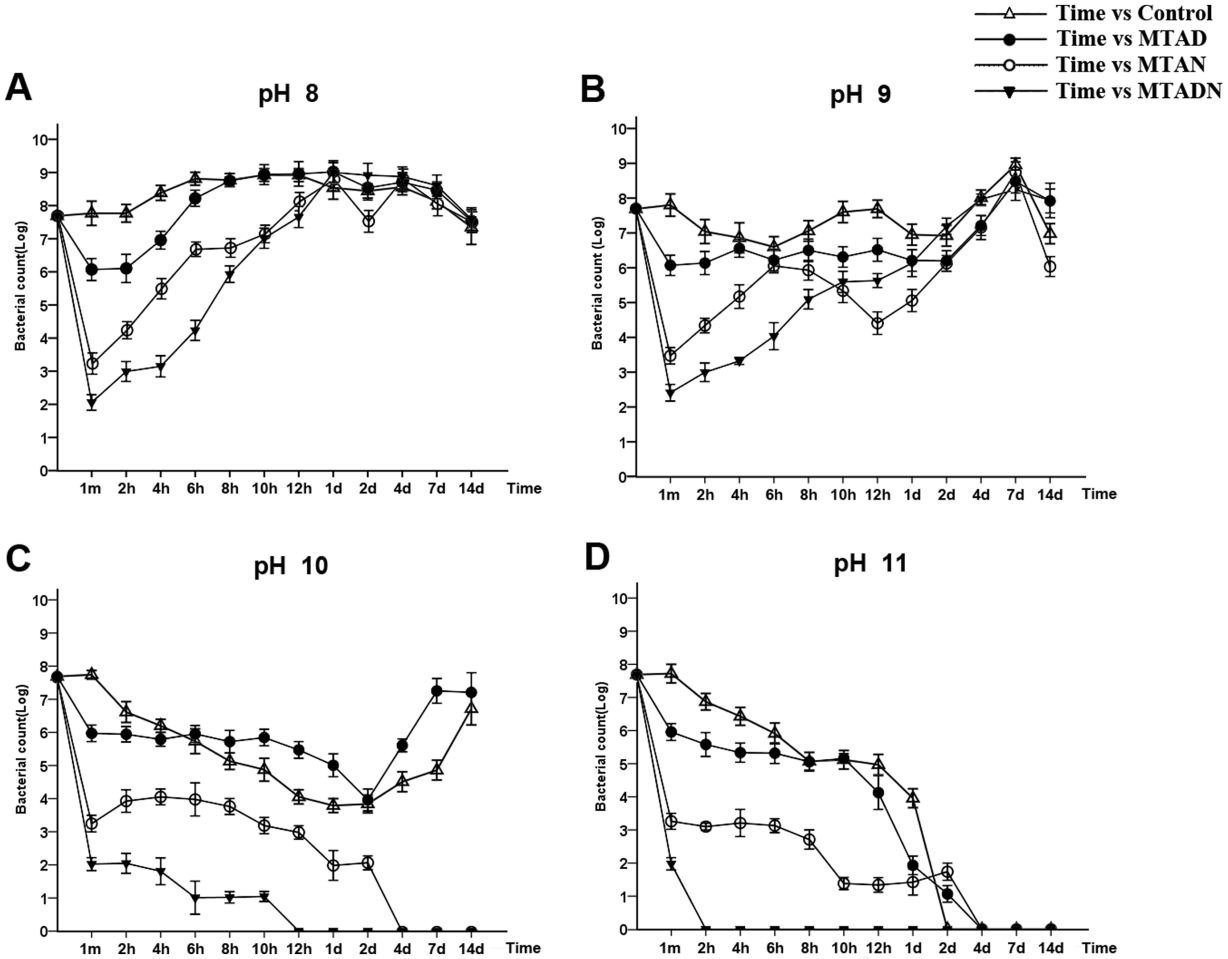
Evaluation of *E. faecalis* resistance to alkaline environments. *E. faecalis* was challenged with pH 8, pH 9, pH 10, and pH 11 conditions after being initially treated with MTAD, MTAN, or MTADN for 1 min. Furthermore, *E. faecalis* without pretreatment with drugs was used as a control. Graphs of the *E. faecalis* survival rates vs. time were plotted. The y-axis represents the logarithmic value of the viable cell concentration (CFU/ml), and the x-axis represents the time of challenge.

Alkaline resistance of *E. faecalis* was evaluated after treatment with three intracanal irrigants, MTAD, MTADN and MTAN, for 1 min. In the pH 8 challenge, *E. faecalis* after the treatment of three irrigants was grown to a maximum in 1 day and subsequently slowly decreased, which is similar to the control group without antibiotic treatment (Fig. 1A). In the pH 9 challenge, the four groups of *E. faecalis* were grown to a maximum in 7 days, and the alkaline solution did not effectively inhibit *E. faecalis* growth (Fig. 1B). In the pH 10 challenge, the survival rate of *E. faecalis* treated with MTAD decreased to a minimum for 2 days and rose to reach a maximum after 7 days. Similarly, *E. faecalis* in the control group also decreased to a minimum for 2 days and to a maximum after 14 days. In the MTADN and MTAN groups, *E. faecalis* was completely killed after challenge in pH 10 solution for 12 h and 4 days, respectively (Fig. 1C). Furthermore, no groups of *E. faecalis* survived in the pH 11 solution, and the times that *E. faecalis* in the MTAD, MTADN, MTAN and control groups was killed was 4 days, 2 h, 4 days, and 2 days, respectively (Fig. 1D).

### Drug resistance of *E. faecalis*

**Figure 2:**
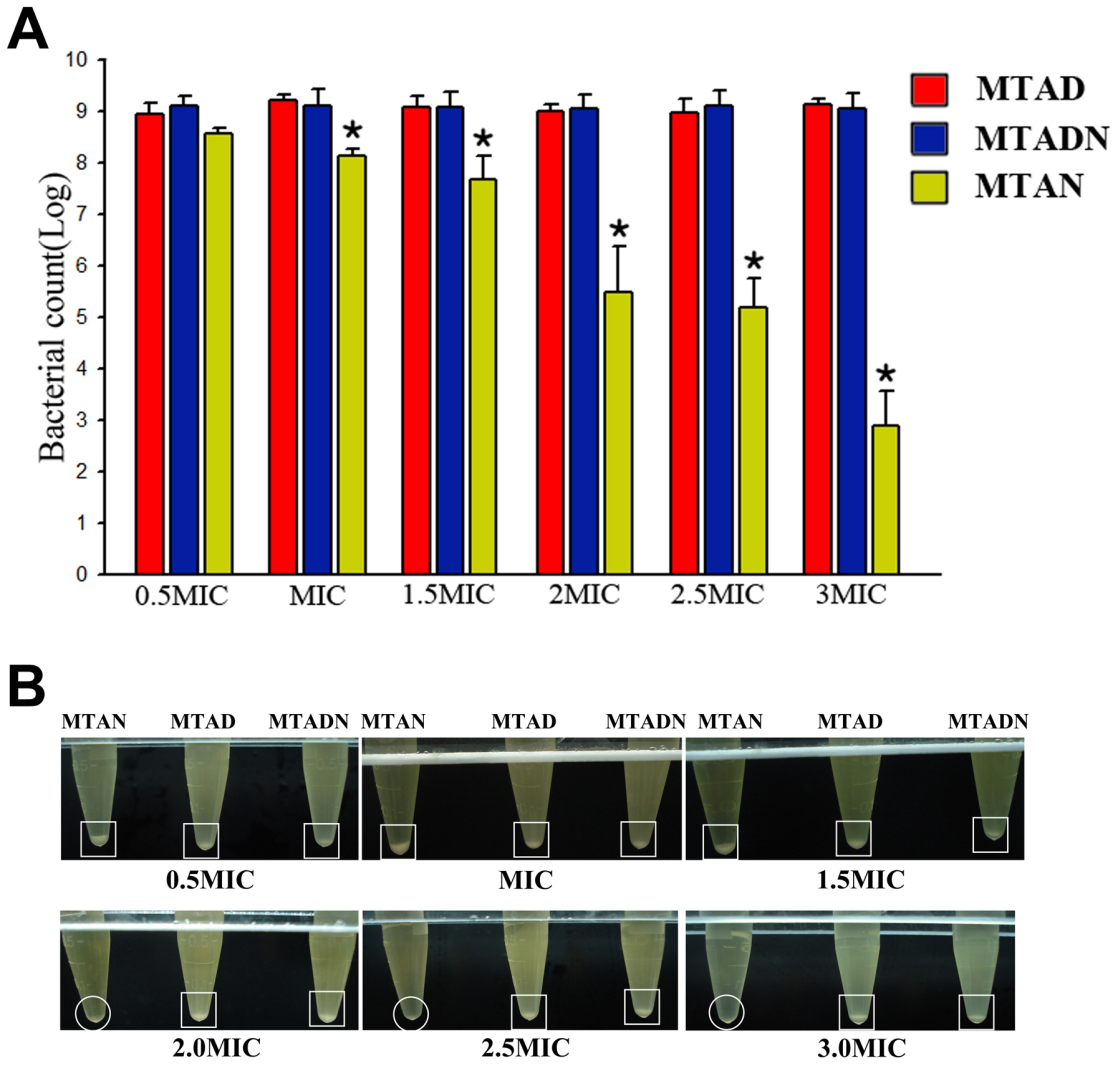
Drug resistance of *E. faecalis* was evaluated by gradually increasing the concentrations of MTAD, MTADN, and MTAN from 0.5 MIC to 3 MIC. (A) Asterisks represent a statistically significant difference between *E. faecalis* survival rates (log10-transformed CFUs counted per milliliter) under treatment with (1) 0.5MIC of MTAN, MTAD and MTADN; (2) MIC of MTAN, MTAD and MTADN; (3) 1.5MIC of MTAN, MTAD and MTADN; (4) 2 MIC of MTAN, MTAD and MTADN; (5) 2.5 MIC of MTAN, MTAD and MTADN; (6) 3 MIC of MTAN, MTAD and MTADN. (B) The bacterial pellet in the eppendorf tube was imaged using a digital camera after treatment with 0.5 to 3.0 MIC of MTAN, MTAD, and MTADN. Square indicated that *E. faecalis* pellets in the bottom of the centrifugal tubes were found after treatment with 0.5 to 3.0 MIC of MTAD and MTADN, while the circles indicated that bacterial pellets were invisible with 2.0, 2.5, and 3.0 MIC of MTAN.

The drug resistance of *E. faecalis* was evaluated by gradually increasing the concentration of drugs. From 0.5 MIC to 3 MIC, *E. faecalis* can survive a higher concentration of MTAD or MTADN after treatment with lower concentrations, whereas in MTAN treatment, the *E. faecalis* survival rate still showed a significant decrease with pretreatment at a low concentration. The *E. faecalis* survival rates were statistically significantly different after treatments of the same concentration of MTAD or MTADN and MTAN (*p*<0.05) (Fig. 2A). Similarly, clear cell pellets were visualized after 0.5 MIC to 3 MIC of MTAD or MTADN, but the pellet was barely visible after 2 MIC or more of MTAN (Fig. 2B).

### Effect of sub-MIC of antimicrobials on pathogenic genes

**Figure 3:**
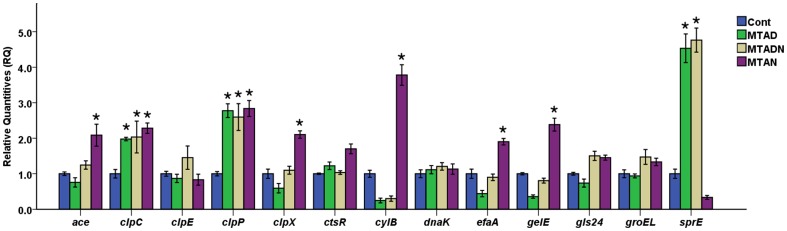
RT-PCR was used to evaluate the effects of sub-MIC of MTAD, MTADN, and MTAN on stress and virulence-associated genes in *E. faecalis*. 16S rRNA was used as an internal control. The asterisk represents a statistically significant difference between the relative quantities (RQ) of gene expression of *E. faecalis* under sub-MIC of MTAD, MTADN, or MTAN and the control.

RT-PCR was used to evaluate the effect of sub-MIC of MTAD and its modifications on stress and virulence-associated genes in *E. faecalis* (Fig. 3). The expression of *clpC* and *clpP* was up-regulated in response to sub-MIC levels of each drug, and there was a statistically significant difference between drugs and control groups (*p*<0.05). Transcriptions of *ace*, *clpX*, *cylB*, *efaA*, and *gelE* were significantly up-regulated, and *sprE* transcription was down-regulated after treatment with sub-MIC of MTAN. However, transcription of *dnaK*, *gls24*, and *groEL* was insignificantly induced by the three drugs. Furthermore, sub-MIC of MTAD and MTADN significantly induced the transcription of *sprE* approximately 5-fold, and a statistically significant difference was found between the two drugs and control groups (*p*<0.05).

## Discussion

PAE and PASME have increasingly become the focus of investigations designed to determine the optimal dosage regimens for antimicrobial agents, and have been employed to evaluate the effect of sub-MIC of antibacterial agents on pathogens [16], [17], [18], [19]. PAE and PASME are dependent on some factors, such as the concentration of the antibiotic, time of antibiotic exposure, and bacterial species [11]. At present, there has been no investigation of the effect of sub-MIC levels of intracanal irrigants on pathogens associated with root canal infections. Our previous studies indicated that nisin can significantly improve the antibacterial activity of MTAD against *E. faecalis* [8], [9]. The determination of PAE and PASME was properly modified according to the potential active time of intracanal irrigant during the root canal procedure. *E. faecalis* was challenged with MTAD and its modifications at the original concentration for 1 min. In the assay of PAE, PASME, and *E. faecalis* resistance to antimicrobials, no significant difference was found between MTAD and MTADN because the previous study indicated that the MIC of 3% nisin alone was at a 1∶32 dilution and the MIC of MTAD was at a 1∶8192 dilution [8]. Thus, 3% commercial nisin in MTADN at a 1∶8192 dilution is too low a concentration to exert its roles. However, this did not indicate that nisin addition did not improve antibacterial role of MTAD. In their original concentration, MTADN is significantly superior to MTAD in some experiments, such as the alkaline resistance assay of the present study and the morphological observation of the previous study [8]. Furthermore, we found that the PASME of MTAN was longer than that of MTAD and MTADN. This might be related to the MICs of each of these drugs. In our previous study, the MIC of MTAN far exceeded that of MTAD and MTADN [8]. Thus, *E. faecalis* showed longer PASMEs when exposed to 0.1, 0.2, and 0.3 MIC of MTAN than when exposed to MTAD or MTADN. However, in the Harada *et al*. study, strains with higher MICs of orbifloxacin and enrofloxacin consistently showed shorter PAE and PASME when compared to those with lower MICs [17]. In another study, two strains of *Staphylococcus aureus* with the same MICs showed different PAE and PASME [20]. Thus, the MIC of antibiotics was not a unique dependent factor for PASME, and the relationship of the MICs of antibiotics with PASME requires further investigation.

*E. faecalis* showed the least resistance to alkaline environments after treatment with MTADN. This result indicated that nisin, in combination with doxycycline, could help calcium hydroxide intracanal dressing to better inhibit the pathogenic bacteria *E. faecalis*. Calcium hydroxide dissociates into calcium and hydroxyl ions on contact with aqueous fluids, and hydroxyl ions cause the lethal effect on bacterial cell by damage to the bacterial cytoplasmic membrane, denaturing of proteins, and damage to DNA [21]. In MTADN and MTAN, the antibacterial peptide nisin exerts its antibacterial activity by forming pores in cell membranes, thus disrupting cell well synthesis and causing a rapid efflux of essential cytoplasmic small molecules [22], [23]. The pores made by nisin facilitate the penetration of hydroxyl ions into bacteria, resulting in rapid death.

Currently, the emergence of bacterial resistance to antibiotic drugs continues to grow, and exposure to nonlethal concentrations of antibiotics is considered to be the major drive in the selection of antibiotic-resistant bacteria [24], [25]. Some antimicrobials peptides have been developed and have entered into clinical trial. The antimicrobials peptide nisin, a component of MTAN or MTADN, has many good properties and is low in toxicity, odorless, colorless, and tasteless. Furthermore, nisin has low drug resistance rates similar to most antimicrobials peptides [26]. In the evaluation of drug resistance, a higher concentration of MTAN still effectively inhibited *E. faecalis*, which was exposed to lower concentrate ons of MTAN. However, *E. faecalis* was still resistant to higher concentrations of MTADN after being pre-treated with lower concentrations, despite the presence of nisin in MTADN. This is due to the different MIC of MTAN and MTADN. A previous study also demonstrated that the MIC of MTADN was a very low concentration (1∶8192 dilution), and 3% commercial nisin in MTADN at such a low concentration hardly exerted its role because the MIC of nisin (1∶32 dilution) was greater than that of doxycycline (1∶8192 dilution) [8]. Furthermore, resistance to tetracyclines was prevalent due to the misuse of tetracyclines in the past [27]. Thus, similar to MTAD, *E. faecalis* are resistant to MTADN at low concentrations due to the unavailability of low concentrations of nisin.

The pathogenic genes tested in the present study had been previously identified and found to affect the pathogenicity of *E. faecalis* [15]. Little is known regarding the use of sub-MIC of intracanal irrigants and their contribution to the switch to pathogenicity in *E. faecalis*. Antibiotics at sub-MIC levels can act as stress inducers or cues/coercion on receiver bacteria and thus induce diverse biological responses [12]. In the present study, sub-MICs of MTAD, MTAN, and MTADN demonstrated different levels of the induction for pathogenic genes in *E. faecalis*. MTAD and MTADN at 0.25 MIC resulted in relatively unchanged expression levels for the tested genes because the active concentration of nisin was very low. However, MTAN had different effects compared to MTAD and MTADN on some genes, even resulting in changes in expression in the opposite direction, such as for the *sprE* gene. This result indicated that *E. faecalis* may enter different stress states in response to doxycycline or nisin. However, the effect of the two different stress states on the pathogenicity of *E. faecalis* still requires further investigation.

Previous studies have indicated that nisin can significantly improve the antibacterial activity of MTAD by either substituting for or acting in combination with doxycycline [8], [9]. The results of the present study indicated that nisin improves the post-antibiotic sub-MIC effects of MTAD against *E. faecalis* as well as sensitizes it to alkaline environments, and thus, nisin addition might be used to further develop the application of MTAD in root canal irrigation.
